# Stem/progenitor cell in kidney: characteristics, homing, coordination, and maintenance

**DOI:** 10.1186/s13287-021-02266-0

**Published:** 2021-03-20

**Authors:** Jiewu Huang, Yaozhong Kong, Chao Xie, Lili Zhou

**Affiliations:** 1 State Key Laboratory of Organ Failure Research, National Clinical Research Center of Kidney Disease, Division of Nephrology, Nanfang Hospital, Southern Medical University, 1838 North Guangzhou Ave, Guangzhou, 510515 China; 2 Department of Nephrology, the First People’s Hospital of Foshan, Foshan, Guangdong China; 3 Bioland Laboratory (Guangzhou Regenerative Medicine and Health Guangdong Laboratory), Guangzhou, China

**Keywords:** Stem/progenitor cells, Kidney, Microenvironment, Therapy

## Abstract

Renal failure has a high prevalence and is becoming a public health problem worldwide. However, the renal replacement therapies such as dialysis are not yet satisfactory for its multiple complications. While stem/progenitor cell-mediated tissue repair and regenerative medicine show there is light at the end of tunnel. Hence, a better understanding of the characteristics of stem/progenitor cells in kidney and their homing capacity would greatly promote the development of stem cell research and therapy in the kidney field and open a new route to explore new strategies of kidney protection. In this review, we generally summarize the main stem/progenitor cells derived from kidney in situ or originating from the circulation, especially bone marrow. We also elaborate on the kidney-specific microenvironment that allows stem/progenitor cell growth and chemotaxis, and comment on their interaction. Finally, we highlight potential strategies for improving the therapeutic effects of stem/progenitor cell-based therapy. Our review provides important clues to better understand and control the growth of stem cells in kidneys and develop new therapeutic strategies.

## Introduction

Chronic renal disease (CKD) has become a public health problem, affecting over 10% of the global population. In the high-risk populations, the prevalence of CKD is up to 50% [1]. Among the etiology of CKD, acute kidney injury (AKI), characterized by a rapid decline of renal function, is considered as a key mediator of CKD and the subsequent end stage of renal disease (ESRD) [2]. However, although renal replacement therapies such as dialysis could be a substitute for sustaining the basal renal function, the repair of kidney itself is the main problem which needs to be solved. Although stem/progenitor cell-based tissue repair and regenerative medicine have been gradually investigated, there are still many areas unexplored. In this review, we summarize the general characteristics of stem/progenitor cells and their homing capacity in kidney. We also highlight the microenvironments involved in stem/progenitor cell maintenance and provide potential strategies for improving stem/progenitor cell functions.

Stem/progenitor cells are a group of specific cells that possess the abilities of self-renewal, multipotent differentiation, and repair after organ injury [3]. Compared with stem cells, progenitor cells display a limited capability of differentiation. The microenvironment could greatly influence their differentiation and self-renewal [4]. Tissue-specific stem cells have been observed in many organs, including kidney, bone marrow, gastrointestinal mucosa, liver, brain, prostate, and skin [4–8]. Stem/progenitor cells can differentiate into epithelial cells, myofibroblasts, and smooth muscle cells in embryonic metanephric mesenchyme [9–11]. The mesenchymal stem cell (MSC) population plays the important role in the embryogenesis of kidney [12, 13]. While in the adult kidney, the two different sources for stem/progenitor cells including resident renal stem/progenitor cells and circulating stem/progenitor cells which are mainly derived from bone marrow, also greatly facilitate the local repair processes through anti-inflammation and immune-modulatory effects [14–17]. There have been some studies showing that stem/progenitor cells could ameliorate kidney injury and improve renal function in ischemia/reperfusion injury (IRI) [3, 5, 15, 18, 19], nephrotic syndrome [20], acute renal failure by intramuscular injection of glycerol [21–23], and an adriamycin-induced model [24].

Circulating stem/progenitor cells include endothelial progenitor cells (EPCs), hematopoietic stem cells (HSCs), and bone marrow-derived MSCs (BMSCs). EPCs, possessing the ability to repair endothelium, are derived from the bone marrow and can be mobilized to the peripheral circulation upon a variety of stimuli [25]. HSCs are a kind of stem cells in the bone marrow, owning the capacity to self-renew, proliferate, and differentiate to replenish the blood and immune systems [26]. HSC transplantation is effective in autoimmune disease [27–29], and also greatly improves renal function in autoimmune nephropathy such as IgA nephropathy [30, 31], focal segmental glomerulosclerosis (FSGS) [32], and crescentic glomerulonephritis [33], by eradicating autoreactive immune cells and regenerating a naive, self-tolerant immune system [34]. A large body of evidences indicate a great of potential therapeutic effects of BMSCs on AKI [35–37], CKD [37, 38], FSGS [39, 40], diabetic nephropathy [41–43], renovascular disease [44], lupus nephritis [45, 46], polycystic kidney disease [47], and others [48–51]. Studies have also shown that EPCs contribute to endothelial repair in IRI-induced kidney [52, 53] and restore the microvasculature, hemodynamics, and renal function in the stenotic kidney [54–56]. To better understand the role of stem/progenitor cells in kidney, we would focus on their characteristics and origin, the mechanism underlying their effects on kidney recovery, and strategies of stem/progenitor cell-based therapy in the following.

## The origin of stem/progenitor cells in the adult kidney

### Kidney-derived stem/progenitor cells

**Fig. 1:**
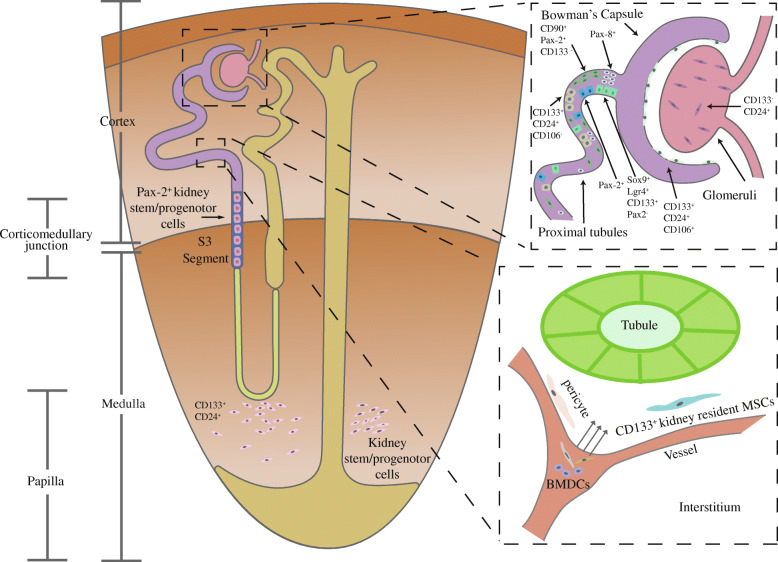
Multiple stem/progenitor cells in kidney, which are located in kidney in situ or originated from circulation, especially bone marrow. Furthermore, there are differences in these kidney-derived stem/progenitor cells considering their location. Stem/progenitor cells in glomeruli are CD24^+^CD133^−^-MSC-like cells. The CD133^+^CD24^+^CD106^+^-stem/progenitor cells are primarily located in urinary pole in Bowman’s capsule. Those cells closer to the urinary pole have more activities than those closer to the vascular pole. CD133^+^CD24^+^CD106^−^ cells are in tubules, especially proximal tubules, with fewer mitochondria and less cytoplasm and without brush border than other tubular epithelial cells. In addition, CD90^+^Pax-2^+^CD133^−^-MSC-like cells, Pax-2^+^ tubule-like cells, and Pax-8^+^ cells also locate in tubules. Notably, Sox9^+^Lgr4^+^CD133^+^Pax-2^−^ cells, primarily located in proximal tubules with epithelial polarity and brush border, could differentiate into proximal tubule, loop of Henle, and distal tubule segments, but not into collecting duct. In S3 segment of nephron, there is a group of Pax-2^+^ stem/progenitor cells, which have the perfect repair capabilities although they have an immature tubular epithelial-like phenotype. Renal papilla is also a niche for renal stem/progenitor cell homing. These CD24^+^CD133^+^ spindle-shaped cells are primarily located in the very outer part of the papilla which is in close proximity to tubules. Besides, there are also pericytes and CD133^+^-kidney-resident MSCs close to the vessel in the interstitium

Many studies have demonstrated kidney-derived stem/progenitor cells in the adult kidney, the majority of which express MSC markers such as CD44, and kidney embryonic stem cell (ESC) markers such as CD24 and Pax-2, but not lineage-specific markers [5, 9, 22, 24, 57, 58], could self-renew and differentiate into mesodermal lineages, including adipogenic, osteogenic, and chondrogenic lineages. There are differences of stem/progenitor cells in different area of the kidney (Fig. 1).

#### Renal stem/progenitor cells in glomeruli

Resident stem/progenitor cells with mesenchymal phenotypes have been found in mouse and human adult glomeruli [59, 60]. These cells can differentiate into mesodermal lineages, endothelial cells, podocytes, and mesangial cells under certain cultural conditions. Different from other kidney-derived stem/progenitor cells, they do not express CD133. These cells not only exhibit a MSC phenotype, but also express ESC markers CD24 and Pax-2 [5, 9, 57], which are negative in BMSCs. It has been found that CD24^+^CD133^+^-MSC-like cells in Bowman’s capsule belong to renal stem/progenitor cells [21, 22], but CD133^+^ cells in glomeruli do not express CD24 and MSC markers and cannot undergo self-renew [59]. To identify the origin of these CD24^+^CD133^−^-MSC-like cells, Bruno et al. isolated them in glomeruli of an explanted kidney from a male donor transplanted into a female recipient, and found that there was no double X chromosome in the 48-selected MSC-like cells. Hence, they believed that these cells were kidney-resident MSCs rather than BMSCs homing to the kidney. Another article shows that although resident kidney MSCs from glomeruli can differentiate into mesodermal lineages, they are different from BMSCs. Compared with BMSCs, resident kidney MSCs exhibit mineralized nodules rather than mineralization of the whole monolayer after differentiating into osteogenic lineages. Besides, the adipogenic differentiation in kidney-resident MSCs seems to be less efficient [60] and also indirectly identified that these stem cells are not derived from bone marrow.

#### Renal stem/progenitor cells in Bowman’s capsule

Many studies have confirmed the existence of CD24^+^CD133^+^ cells in Bowman’s capsule, especially in the urinary pole of Bowman’s capsule. Compared with all other parenchymal cells of the kidney, they show higher resistance to injurious agents [20, 21, 61–63]. We can distinguish their source because renal stem/progenitor cells in Bowman’s capsule express CD106, but stem/progenitor cells in the tubules do not. Besides, CD133^+^CD24^+^CD106^+^ cells exhibit a higher rate of proliferation than those with negative expression of CD106. These cells with CD133^+^CD24^+^CD106^+^ expression prefer to differentiate toward the phenotypes of podocyte and the tubular lineage. By contrast, CD133^+^CD24^+^CD106^−^ cells mainly prefer to the tubular lineage differentiation [21]. CD133^+^CD24^+^CD106^+^ cells are primarily located in the urinary pole of Bowman’s capsule, while CD133^+^CD24^+^CD106^−^ cells are mostly expressed in proximal tubules, so they are close to each other. The abilities of self-renewal and differentiation of CD133^+^CD24^+^CD106^−^ cells are less than CD106^+^ cells. However, they both express vimentin, cytokeratin 7, and cytokeratin 19, highlighting the similarity between the two cells [64]. CD133^+^CD24^+^CD106^−^ cells may derive from CD133^+^CD24^+^CD106^+^ cells and this represents a more committed step toward complete differentiation into the tubular lineage [21].

Renal stem/progenitor cells in Bowman’s capsule are a special type of parietal epithelial cells, which exhibit a high potential of self-renewal and multilineage differentiation and express kidney ESC as well as MSC marker CD44, but not lineage-specific markers [20, 22, 65]. These cells also express the stem cell-specific transcription factors Oct-4 and Bmi-1 [22]. Oct-4 is normally expressed in ESCs, for maintaining their immature state, and is required for the pluripotency of germ cells [66]. Bmi-1 is a critical factor in the maintenance of the self-renewal ability of adult stem/progenitor cells. Knockout of Bmi-1 in renal stem/progenitor cells would result in their apoptosis and decrease in their capacity of self-renewal [67]. Notably, the abilities of self-renewal and differentiation are different considering the location. Those cells closer to the urinary pole of Bowman’s capsule have more abilities of differentiation and proliferation than those closer to the vascular pole [20]. Because renal stem/progenitor cells in Bowman’s capsule express kidney ESC markers, thus they are also believed as residual kidney stem/progenitor cells rather than BMSCs.

#### Renal stem/progenitor cells in tubules and interstitium in cortex

There are stem/progenitor cells in tubules, especially proximal tubules [5, 11, 21, 24, 64, 68]. Most of them are capable to differentiate into tubular epithelial cells and even could differentiate into mesodermal lineages such as adipogenic, osteogenic, and chondrogenic lineages. But it still has differences. A study shows that these cells express renal ESC markers such as Pax-2 and have a spindle-shaped morphology. These cells have a positive expression of CD90 and CD44, but are CD133-negative [5]. Other studies show that stem/progenitor cells in tubules are Pax-2 as well as some MSC marker-positive, although there is no morphologic difference between them and other tubular cells [11]. A study also shows that they express MSC markers of CD44 and renal stem/progenitor cell marker Pax-8. They have a strong ability of self-renewal and differentiation into tubule epithelial cells. Interestingly, they could also be induced to differentiate into mesodermal lineages in vitro as well [24].

Most studies have shown that there are CD24^+^CD133^+^-stem/progenitor cells in the tubules, which can regenerate tubular cells and improve renal function after kidney injury [21, 63, 64, 69]. They own the capacities of self-renewal and differentiation into tubular cells [21]. Although they are Pax-2 and CD44 negative, they could express vimentin, cytokeratin 7, and cytokeratin 19, none of which are expressed in the differentiated proximal epithelial cells [63, 64]. What is more, compared with tubular epithelial cells, they have fewer mitochondria and less cytoplasm and have no brush border. Some researchers think that there is also a possibility that this phenotype is the result of the loss of the brush border because of the dedifferentiation of these cells toward a more mesenchymal phenotype. As a result, these cells could be commonly mistaken as renal stem/progenitor cells in tubules [63, 69].

It has been found that Sox9^+^ cells are in adult kidney, which own the high capacity of proliferation and mesodermal lineage differentiation [70]. These stem/progenitor cells are primarily located in proximal tubules, and they have epithelial polarity and brush border [68]. These cells express CD133 and Lgr4, the markers of progenitor cells, but have a negative expression of Pax-2 or common MSC markers. They could differentiate into proximal tubules, loop of Henle, and distal tubule segments, but not into collecting ducts. Sox9^+^ cells are found in the early stage of kidney development and disappear quickly after birth. They possess the high ability of proliferation and are the predominant contributor to repair in tubules after kidney injury. Because most of epithelial cells except those in collecting ducts and glomeruli are descendants of Sox9^+^ cells in the kidney, the studies have different arguments about the increase in Sox9^+^ cells after kidney injury. They think that although most descendants of Sox9^+^ cells no longer express Sox9 gene in normal kidneys, it is activated after kidney injury. The researchers think that de novo activation of Sox9 rather than the expansion of the resident Sox9^+^ population contributes more to the recovery of kidney [68, 70, 71].

Pax-2^+^ cells have been found in the S3 segment of the nephron, characterized with an immature phenotype of tubular epithelial cell and the expression of progenitor and mesenchymal cell markers. These cells have the abilities of self-renewal, differentiation, and tissue repair. They can reconstitute three-dimensional nephron-like structure, including glomeruli, proximal tubules, the loop of Henle, distal tubules, and collecting ducts, but not vasculature. They could also migrate into injured areas and differentiate into mature tubular epithelial cells in vivo [3, 72, 73].

CD133^+^ cells with MSC and kidney ESC markers are located in the interstitium in adult kidney cortex. These cells could differentiate into epithelial or endothelial cells and grow into tubular structures or functional vessels, but they have limited ability of self-renewal [10]. Because they do not express the hematopoietic markers CD34 and CD45, they might be of kidney origin. However, it has also been proposed that these cells may originate from a bone marrow-derived population, which has homed to the kidney a long time ago. Hence, they have lost their markers of hematopoietic lineage.

#### Renal stem/progenitor cells in papilla and interstitium of the medulla

The renal papilla is a niche for adult renal stem/progenitor cells [15, 18, 74–76]. These CD24^+^CD133^+^ spindle-shaped cells co-express MSC markers such as stem cell antigen-1 (Sca-1) and epithelial proteins, have high activity of telomerase, and can differentiate into mesodermal lineages and endothelial cells [15, 18]. These cells are mainly located in the very outer part of the papilla, in close proximity to the tubules, and some are adjacent to the tubular basal surface. These cells can also be found in the cortex and medulla to a less extent [15]. After kidney injury, they proliferate and migrate into injured area to repair tubules, although their generative capacity is restricted.

Lee et al. also found that there are some spindle-shaped cells with kidney ESC markers in the interstitium of the medulla. These cells could differentiate into endothelial, osteoblastic, and tubular epithelial lineages in vitro. Moreover, they are able to differentiate into endothelial cells and tubular cells and preserve renal function after ischemic renal injury [19].

#### Remaining embryonic kidney stem/progenitor cells

Renal progenitor cells in human embryonic kidney express CD24 and CD133 and have the capacities of self-renewal and multi-lineage differentiation. Like most renal stem/progenitor cells, these cells express MSC and kidney ESC markers, but not hematopoietic markers such as CD45. They construct the human primordial nephron in the early stage, but disappear progressively during nephron development, while the remnant kidney ESCs which locate primarily in the urinary pole of Bowman’s capsule represent < 2% of whole cells in the adult kidney [23]. However, these cells can differentiate into many kinds of kidney-resident cells and even into mesodermal lineages. After AKI, renal progenitor cell administration could enhance tissue repair and induce the recovery of renal function as well as structure. Because most renal stem/progenitor cells exhibit a similar phenotype to embryonic kidney stem/progenitor cells, renal CD24^+^CD133^+^ stem/progenitor cells in the adult kidney may all be derived from renal ESCs [23].

#### Renal stem/progenitor cells and kidney-resident MSCs

Resident MSCs have also been isolated from adult kidneys. Their characteristics are similar to those of ESCs. These cells are able to differentiate into a wide variety of lineages, including mesodermal lineages, endothelial cells, and erythropoietin-producing fibroblasts. After kidney injury, they migrate into the kidney and promote the recovery of renal function [77–79]. Some researchers believe that the MSC-like renal stem/progenitor cells in embryonic and adult kidneys are merely resident MSCs in the kidney, including in glomeruli, tubules, interstitium, and papilla [13]. Besides, it has been proposed that kidney-resident MSCs are derived from perivascular cells [60], which would explain why renal stem/progenitor cells can be isolated from many parts of the kidney and their MSC-like appearance.

Pericytes, which are vascular mural cells with a function of angiogenesis in kidney [80], modulate the endothelial phenotype and the extracellular matrix composition to stabilize vessels. Mesangial cells are described as the glomerulus-specific pericytes [81]. Of note, some markers of pericytes such as CD146 and CD73 are also expressed in MSCs [13]. Hence, pericytes, exhibiting the potential of mesodermal lineage differentiation, are thought to be renal stem/progenitor cells and considered as resident MSCs around capillary walls [13, 82–84].

Some Gli1^+^ cells around the vasculature expressing the typical MSC markers are considered as immature pericytes. They possess mesodermal differentiation capability in the kidney, and contribute greatly to kidney fibrosis. It has been revealed that around 45% of myofibroblasts in the kidney are derived from these Gli1^+^ MSC-like cells [80]. Another study has also shown that pericytes are the main source of myofibroblasts in the kidney [85]. These suggest stem/progenitor cells may also have the bad side effects besides repair.

### Circulating bone marrow-derived stem/progenitor cells homing to the adult kidney

Bone marrow-derived stem/progenitor cells (BMDCs) can be released from bone marrow into the peripheral blood and then move into the injured area to improve the renal function after attracted by a variety of growth factors and inflammatory cytokines released from the injured area [53, 86–90]. It has been reported that in male patients who have received a kidney transplant from a female donor, there are some BMDCs with a Y chromosome in the kidney with the expression of a tubular epithelial cell or podocyte phenotype. This demonstrates that circulating BMDCs can home to the kidney and differentiate into tubular epithelial cells and podocytes [91]. Imasawa et al. also found that after tail vein injection of enhanced green fluorescent protein (EGFP)-labeled BMDCs and subsequent sufficient perfusion with PBS to remove circulating EGFP^+^ cells in glomeruli, the remaining EGFP^+^ cells exhibit several characteristics and markers of glomerular mesangial cells. The numbers of which increase in a time-dependent manner, suggesting BMDCs own the ability to migrate into the kidney and transdifferentiate into mesangial cells after kidney injury [92].

It is reported that BMDCs can fuse with somatic cells [93, 94], which can also lead to the presence of BMDC markers and somatic cell markers in the same cells. However, these studies cannot elucidate whether the endothelial cells, tubular epithelial cells, podocytes, and glomerular mesangial cells detected in this study arise from transdifferentiation or cell fusion [91, 92, 95–99]. In order to answer this question, a study performed the transplantation of bone marrow from female mice into male Fah^−/−^ mice. The presence of the host marker Y chromosome in Fah^+^ tubules, the donor marker, would indicate cell fusion. The study shows that at least half of the bone marrow-derived tubular epithelial cells are generated by cell fusion. However, Fah^+^ Y^−^ tubular epithelial cells may also be generated by cell fusion, rather than from direct transdifferentiation of BMDCs, because it may be the result of decreased division, loss of the Y chromosome, or the artificial limitations of tissue section analysis [100].

After administration of male mouse HSCs into female ischemic mice, there are some cells exhibiting a renal proximal tubular cell phenotype and carrying a Y chromosome, indicating that HSCs could be recruited and transdifferentiate into tubular epithelial cells [87, 101, 102]. Another study shows that HSCs can also transdifferentiate into glomerular mesangial cells [103]. Because the frequency of cell fusion is rare per 10^6^ bone marrow cells and the number of HSC-derived cells greatly exceeds the frequency of cell fusion, the researchers believe that HSCs are unlikely to be involved in the cell fusion, although it cannot be completely excluded [87]. Ikarashi et al. found that after administration of EGFP^+^-bone marrow cells in progressive glomerulosclerosis rat model, some glomerular endothelial cells express the endothelial cell markers PECAM-1 or RECA-1 with the colocalization of EGFP, suggesting the involvement of EPCs in glomerular endothelial cell turnover [104]. Other studies also show that EPCs in the injured kidney could differentiate into endothelial cells and contribute to the rebuilding of glomerular capillaries [89, 105–107]. Researchers believe that EPC-derived cells are prone to transdifferentiate rather than cell fusion, because cell fusion is a very low-frequency event. The numbers of EPC-derived cells greatly exceed the frequency of cell fusion. Furthermore, cell fusion would result in the loss of cell function and lower expression of EGFP, which is contradictory with its significant therapeutic effects [104]. Ezquer et al. found that after tail vein injection of EGFP^+^-BMSCs, they exert a renoprotective effect on diabetic nephropathy mice. EGFP^+^-BMSCs are found in the kidney of diabetic mice while they are undetectable in normal mice, suggesting that the injured kidney could recruit BMSCs [41]. Another study shows that after transplanting the bone marrow of EGFP-positive rats into wild-type rats, BMSC transdifferentiate into mesangial cells to provide structural support for glomerular capillaries [108]. Other studies also show that BMSCs are able to transdifferentiate into podocytes, mesangial cells, tubular epithelial cells, etc. both in vitro or vivo [88, 109–111]. Although cell fusion is a low-frequency event and it is contradictory with the significant therapeutic effects, it cannot be completely excluded considering it as a repair mode.

## Interaction between stem/progenitor cells and kidney microenvironment

**Fig. 2:**
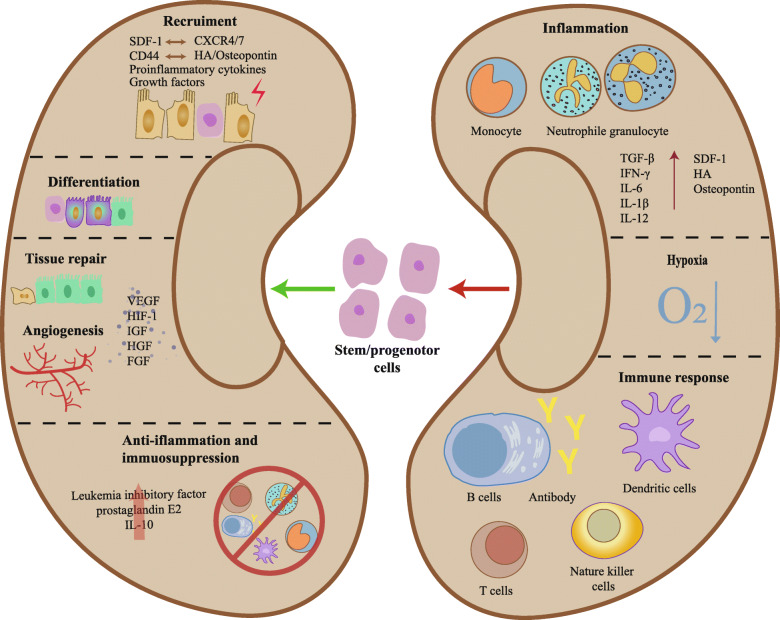
After kidney injury, the microenvironment would turn to be inflammatory, hypoxic, and immunostimulatory. The proinflammatory microenvironment induced by neutrophil granulocyte and mononuclear macrophage infiltration leads to the massive release of injurious factors such as TGF-β, IFN-γ, IL-6, and so on. This will recruit stem/progenitor cells through the interaction between SDF-1 and CXCR4/7, HA/osteopontin and CD44, and others. In addition, the insufficient oxygen supply caused by ischemia and increase in oxygen consumption in the disease state would certainly result in the hypoxic microenvironment. This would aid to the recruitment and differentiation of stem/progenitor cells and induce the production of lots of angiogenesis factors in these cells to facilitate tissue repair. The immune responses include the activation of B cells, T cells, NK cells, and dendritic cells, which constructs the local immune microenvironment to affect the stem/progenitor cell-induced tissue repair. Overall, the various factors in the local microenvironment build up an intricate network to cooperatively assist stem/progenitor cell functions and finally promote stem/progenitor cell-dependent tissue repair through their beneficial effects on angiogenesis, anti-inflammation, immunosuppression, and others

After kidney injury, kidney cells could release a variety of growth and inflammatory factors, including insulin-like growth factor-1 (IGF-1), hepatocyte growth factor (HGF), basic fibroblast growth factor (bFGF), and vascular endothelial growth factor (VEGF), to promote tubule regeneration and kidney repair [112–115]. Moreover, it has been reported that renal stem/progenitor cells, resident MSCs, and BMDCs could self-renew, migrate into the injured area, and then differentiate to aid tissue repair [11, 18, 21, 73, 78] (Fig. 2).

### Microinflammation

#### Recruitment of stem/progenitor cells

The stromal-derived factor-1 (SDF-1)/chemokine (C-X-C motif) receptor 4 (CXCR4) axis plays an important role in the migration of BMDCs and renal stem/progenitor cells [116, 117]. SDF-1, a main regulator of migration and mobilization for BMDCs [118], is upregulated in the surrounding resident cells of necrotic area [14, 119, 120]. CXCR4 and CXCR7, the receptors of SDF-1, are highly expressed in renal stem/progenitor cells [119, 121]. CXCR4 is essential for migration, and CXCR7 plays a significant role in adhesion to endothelial cells and survival of kidney stem/progenitor cells [119]. CXCR4 and CXCR7 are also expressed in BMDCs [118, 122–125]. Studies also show that the upregulation of SDF-1 in injured areas increases the expression of CXCR4 in BMSCs [126, 127]. Moreover, the role of CXCR4 or CXCR7 in BMSCs is similar to that in renal stem/progenitor cells [128]. A study shows that the SDF-1/CXCR4 axis plays an important role in BMSC migration as well as in survival and cytokine secretion in the injured area by activating the Akt and Erk pathways [127].

CD44–hyaluronic acid (HA) interaction also plays an important role in the migration of BMDCs to the injured area [129–132]. HA is the major ligand of CD44, which is expressed in BMSCs, will increase after tissue injury in both chronic and acute kidney injury [129, 130, 132]. A study shows that CD44–HA interaction also plays an important role in the stimulatory effects of SDF-1 on BMDC migration [132]. In addition, osteopontin is also upregulated after kidney injury [133, 134], which promotes the expression of its receptor integrin β1 in BMSCs, and leads to the migration of BMSCs in a dose-dependent manner [117, 135]. Moreover, it has been found that CD44v6, another receptor of osteopontin, which is also expressed in BMSCs, may also play an important role in the migration of BMSCs to the injured kidney [135, 136]. The capacity of BMSCs to reshape themselves, depending on their stiffness, is related to the structure of the cytoskeleton and significant for migration due to the physical ability when crossing tissue and vessels [137]. Osteopontin also lowers the expression of cytoskeleton proteins through FAK/ERK1/2 pathway, contributing to BMSC migration by reducing cell stiffness [117].

Besides, growth factors and proinflammatory cytokines released by the injured area, including bFGF, VEGF, platelet-derived growth factor (PDGF), transforming growth factor β1 (TGF-β1), IGF-1, HGF, tumor necrosis factor-alpha (TNF-α) [138–140], and interferon-gamma (IFN-γ) [140], also play a significant role in the migration of BMSCs [117, 141]. However, the sustained upregulation of PDGF, a powerful growth factor in BMSC recruitment and tissue repair in the injured kidney [142], could also lead to renal fibrosis by activating myofibroblasts, mesangial cells, or smooth muscle cells [143]. It has also been found that FGFs, a factor playing an important role in stem cell self-renewal [144], is released after kidney injury to be the requisite for the recruitment of kidney stem/progenitor cells and maintenance of cell adhesion [145].

#### Anti-inflammation and tissue repair of stem/progenitor cell

Differentiation is not the only mechanism by which renal stem/progenitor cells or BMDCs repair the injured kidney; this is also accomplished through a paracrine mechanism. It has been found that extracellular vesicles (EVs) could form an important part of the paracrine system. EVs are small, lipid membrane-enclosed subcellular structures carrying biomolecules of proteins, lipids, nucleic acids, and sugars. They are released from cells into the extracellular environment and even could reach remote areas. EVs include exosomes, microparticles, or microvesicles [146–149]. Notably, kidney stem/progenitor cells could secrete IL-15, endothelial growth factor, HGF, leukemia inhibitory factor, inhibin-A, decorin, VEGF, and recombinant human bone morphogenetic protein (BMP)-7 through direct release or through shuttling mRNA or miRNA using EVs, to repair renal injury, alleviate inflammation, and retard fibrosis [14, 150–152]. A study shows that the effects of EVs of kidney stem/progenitor cells may primarily depend on the shuttling of mRNA or miRNA, because after treatment with RNase, EVs are not effective on improving kidney function and aiding recovery. Meanwhile, physiological doses of RNase cannot degrade the RNA in the EVs, but high-dose can [152].

Besides, BMSCs also play a significant role in anti-inflammation and facilitating tissue repair after kidney injury. Several studies have suggested that the main protective mechanism of BMSCs in kidney is through paracrine action rather than differentiation [153–156]. They performed the study using the Y chromosome as a marker of donor BMSCs; they could not find BMSCs within the tubules in that infusion of BMSCs, and BMSCs were rare in the renal interstitium. However, they found kidney failure was ameliorated [155].

Moreover, conditioned medium from cultured BMSCs not only induces migration and proliferation of renal epithelial cells and greatly alleviates proximal tubular cell death in vitro, but also inhibits kidney injury after intraperitoneal administration [155]. BMSC administration downregulates TGF-β, IFN-γ, IL-6, and IL-1β expression and further represses inflammation and fibrosis through the direct secretion of repairing cytokines or release of EVs [14, 42, 153, 157–159]. The administration of BMSCs also inhibits the expression of apoptosis-related proteins such as Bax, cytochrome c, and caspase-3, increases the activity of superoxide dismutase (SOD), and regulates autophagy-associated proteins such as Beclin 1, PINK1, Parkin, p-Parkin, LC3B, and MAPK signaling-related proteins to decrease apoptosis and oxidative stress [160–163]. However, one study also shows BMSC could differentiate into myofibroblasts upon long-term stimulation by TGF-β [164].

### Hypoxia and angiogenesis

Hypoxia is one of the most common features of tissue injury [159]. A study shows that hypoxic microenvironment could enhance the migration of BMSCs [141]. The expression of SDF-1 in kidney is increasing after ischemic or hypoxic injury [120, 128, 165]. Besides, hypoxia also increases the expression of CXCR4 in BMSCs [128]. It indicates that hypoxia may play a significant role in the recruitment of stem/progenitor cells into injured kidney by SDF-1/CXCR4 axis. After renal stem/progenitor cells migrating into the injured area, the microenvironment of low-oxygen tension induces them to proliferate and produce erythropoietin to limit renal fibrosis via activating the hypoxia-inducible factor-2α (HIF-2α) axis by prolyl hydroxylase [76, 166, 167]. Erythropoietin could also increase the expression of SDF-1 in kidney [168]. Hence, the interaction between hypoxic microenvironment and renal stem/progenitor cells may form a positive cycle for recruiting stem/progenitor cells and subsequent repair.

Kidney-resident MSCs could release the EVs carrying VEGF, bFGF, and IGF-1, the proangiogenic factors, to contribute repair through their anti-apoptotic and angiogenic effects [152, 169, 170]. Hypoxic culture of MSCs could induce the secretion of these pro-vasculogenic factors [159], such as IGF-1, VEGF, bFGF, HGF, and thymosin β4 (TB4), to facilitate tissue repair and ultimately promote kidney protection [51, 113, 154, 171–174]. Similar to kidney-resident MSCs, studies have shown that BMSC-derived EVs could also protect against kidney injury through anti-apoptotic and angiogenic effects [159, 175]. The biological effects of BMSC-derived EVs may mainly depend on the contained RNA, including mRNA and microRNA, because RNase could abolish the effects of EVs [176]. The EVs, as a tool of transportation, can shuttle the specific subset of cellular RNAs of BMSCs, especially RNAs associated with transcription and proliferation, to modulate energy metabolism and cellular pathways of recipient cells [35, 169, 176–178]. Studies have shown that more EVs are engrafted into the injured kidney than the normal after injection. Furthermore, the majority of EVs are taken up by tubular epithelial cells and peritubular capillaries, but some also by glomeruli [169]. However, the underlying mechanism is still a mystery.

A study shows that EPCs could be mobilized into glomeruli after kidney injury. They would self-renew, differentiate into glomerular endothelial cells, and express hypoxia-inducible factor 1 (HIF-1), the key transcription factor driving VEGF expression [54, 179], to rebuild the glomerular capillary structure [89, 180]. Moreover, EPCs can also enhance renal growth factor expression and retard oxidative stress in ischemic kidney [181]. Like MSCs, EPCs can also ameliorate kidney injury and enhance angiogenesis through EVs release for delivering miRNA, because some researches show the renoprotective effects of EVs are lost after treatment with RNase or specific miRNA-antagomirs [182–184].

### Local immune response

Studies have shown that after kidney ischemia injury, mature dendritic cells are increased. As an antigen-presenting cell, dendritic cells would induce T cell proliferation and migration to inspire the immune response [185]. T cells, especially CD4^+^-T cells, are an important source to persist inflammation in CKD patients [16, 186, 187]. Abnormal activation of T cells leads to a release of proinflammatory cytokines such as TNF-α and IFN-γ [186], which play an important role in the recruitment of BMSCs [138–140]. B cells may also play a role in kidney injury. A study shows that B cell deficiency plays a protective role in renal IRI mice [185]. The network of dendritic cells, T cells, and B cells constructs the local immune microenvironment to affect the stem/progenitor cell-induced tissue repair.

Studies show that MSCs and renal stem/progenitor cells in papilla exhibit the capacity of immunomodulation [15, 16, 74]. They can greatly reduce T cell proliferation through cell-cell contact and inhibit the stimulatory effects of dendritic cells on T cells and the secretion of prostaglandin E2, an anti-inflammation factor [15, 16, 188, 189]. BMSCs also exert an inhibitory effect on the proliferation of T cells and natural killer cells, inhibit alloantigen recognition and processing of dendritic cells, and modulate B cell functions, including proliferation and antibody production to trigger immunosuppression [16, 17, 190–194]. Studies have shown that BMSCs could not only inhibit dendritic cell maturation, but also inhibit the antigen-presenting function by inhibiting their migration into lymph nodes, downregulating IL-12 expression, and upregulating IL-10 expression [195–197]. BMSCs also inhibit the cytotoxic activity of NK cells by decreasing NKp30 and natural killer group 2 and downregulating member D, the receptors for natural killer cell activation, and target-cell killing [198]. BMSCs also contribute to the transition of T cells from a proinflammatory state to an anti-inflammatory state and inhibit the formation of cytotoxic T lymphocytes [198, 199], which may partly explain the kidney protective function of BMSCs in autoimmune nephropathy. Compared with T cells, the influence of BMSCs on B cells is controversial. Some studies have shown that BMSCs can inhibit B cell proliferation, differentiation, and chemokine secretion, whereas other studies have shown that BMSCs could promote the proliferation and stimulate the secretion of antibodies [198].

## Strategies of stem/progenitor cell therapy for kidney injury

### Preconditioning

After kidney injury, BMSCs, EPCs, HSCs, and kidney stem/progenitor cells migrate into the injured area, but the local microenvironment may also lead to their apoptosis because of ischemia, inflammation, deficiency of oxygen and nutrition, and the upregulation of oxidative stress as well as immunological rejection. Studies have shown that the function of EPCs and BMSCs in CKD is greatly impaired [200–202]. Undoubtedly, the efficacy of stem/progenitor cells primarily depends on their ability to migrate into injured areas and their survival time. A strategy to improve stem cell homing and survival seems necessary.

Stem cell preconditioning is a promising way for us. These strategies include incubation or co-injection with cytokines or chemical compounds, hypoxia stimulation, and genetic modification [203]. Hypoxic or ultrasound preconditioning, incubation with TGF-β1 or IGF-1, and co-injection with erythropoietin can increase the expression of CXCR4 in BMSCs, further promoting BMSC homing to the kidney through the CXCR4/SDF-1 axis [128, 168, 204–210]. Leflunomide also increases mobilization of BMSCs, HSCs, and EPCs in the peripheral blood and promotes their migration into the injured kidney [211]. Hypoxic preconditioning upregulates the expression of CXCR7 in BMSCs [212], and not only improves BMSC chemotaxis but also enhances secretion of therapeutic soluble factors, such as VEGF, IGF-1, HGF, FGF, and angiopoietin and increases cell viability in injured area [128, 159, 213]. Preconditioning with sevoflurane can also produce protective effects on BMSC survival by minimizing apoptosis and recovering the loss of mitochondrial membrane potential [214]. Erythropoietin can not only enhance the proliferation and kidney protective function of BMSCs [215, 216], but also protect the kidney by enhancing mobilization and recruitment of EPCs [217, 218]. Statin pretreatment ameliorates oxidative stress, inhibits the inflammatory response in the injured kidney, and increases the survival of implanted BMSCs [219], and also increases EPC recruitment and reduces apoptosis [220]. Pretreating BMSCs with melatonin enhances their survival after migration into the injured kidney [221, 222]. Administration of pioglitazone could improve the function of MSCs and EPCs by reducing endoplasmic reticulum stress and mitochondrial fusion [223–226]. Pretreatment with the dipeptidyl peptidase-4 inhibitor sitagliptin, an agent for incretin-based therapies for type 2 diabetes [227], can enhance EPC mobilization by increasing plasma SDF-1α concentrations, possibly be an effective strategy for the treatment of diabetic nephropathy [228].

Genetic modification is also a useful way of preconditioning to enhance stem cell homing to the kidney. CXCR4-overexpressing BMSCs constructed by lentivirus infection have a stronger ability of homing to the kidney and enhanced paracrine actions to produce HGF, BMP-7, and the anti-inflammatory cytokine IL-10 [229]. Kallikrein-modified BMSCs through lentivirus infection also have stronger anti-oxidative, anti-apoptotic, anti-inflammatory, and angiogenic effects on kidney injury [230]. MiR-let7c-overexpressing BMSCs can deliver more miR-let7c through EVs to injured kidneys, further reducing the expression of fibrosis-related genes and renal fibrosis [231]. MiR-126-overexpressing BMDCs have an enhanced ability of mobilizing into injured areas by regulating the CXCR4/SDF-1 axis [232].

### Application of bioactive molecules secreted by stem/progenitor cells

As described above, many studies indicate that the bioactive molecules secreted by stem/progenitor cells also play an important role in restoring renal function. Their application could have multiple advantages in clinical applications, including the prevention of stem/progenitor cells from directly exploring the injured microenvironment and an easier productive and storage process [233]. The function of stem cells in CKD is impaired, and allogenic stem cells may be rejected, so the use of bioactive molecules secreted by stem cells is a potential strategy to overcome this problem. Theoretically, cell-free therapies may exhibit superior safety compared with direct delivery of stem cells. BMSC-derived conditioned medium promotes the regeneration of injured kidney tissue, reduces renal inflammation and fibrosis, and restores the microvascular structure in unilateral ureteral obstruction (UUO), 5/6 nephrectomy, and diabetic nephropathy models [48, 233–236]. The effect of EVs from BMSCs on the recovery of kidney is similar to administration with BMSCs, so the application of EVs is also a potential strategy for us. Studies show that allogenic kidney-resident MSC-derived EVs can decrease apoptosis, enhance tubular proliferation and tubule formation, and reduce inflammatory cell infiltration in IRI and UUO models [170, 237]. Besides, both autologous and allogenic BMSC-derived EVs can improve renal function in IRI, drug-induced nephropathy, UUO, and subtotal nephrectomy models [169, 177, 178, 238–241].

### Biomaterials

Biomaterials, which can improve the migration of stem/progenitor cells, enhance their function, and provide a favorable microenvironment, should also be taken into consideration [242]. As described above, bioactive molecules secreted by stem cells exhibit many advantages in restoring renal function, but they are unstable and are rapidly degraded in vivo. To maintain a certain blood concentration, a multiple-dose protocol is required. Biomaterials such as hydrogel, which ensures controlled release of bioactive factors, can solve this problem [243]. EVs also require a frequent dosing because they are rapidly cleared from the body by the reticuloendothelial system after injection into the circulation [244]. Preconditioning with biomaterials is a promising strategy to overcome rapid clearance. Combining or wrapping EVs in a biomaterial matrix can maintain their bioavailability after administration, permitting sustained and controlled release, to enhance therapeutic efficacy [159]. Hydrogels, especially modified hydrogels, could enhance the retention and stability of EVs [245]. A study shows one kind of mesoscale nanoparticles could package small molecules and even large biomolecules such as DNA which is not dependent on the encapsulated cargo and exhibit 26-fold renal selectivity without side effects such as immune reactions as well as liver or kidney impairment [246]. Preconditioning with biomaterials can also enhance stem cell survival, engraftment, and homing. Injectable biomaterials such as hydrogels could increase the retention of stem cells after transplantation [242]. MSC spheroids entrapped in Arg-Gly-Asp-modified alginate hydrogels exhibit decreased apoptosis and increased survival as well as VEGF secretion after transplantation [247]. In addition, the fate of stem/progenitor cells homing to injured areas mainly depends on the local microenvironment. Biomaterials could provide a stem cell niche-like microenvironment for transplanted stem/progenitor cells in vivo [159]. Preconditioning of pro-survival peptides with a slow-releasing of collagen matrix can enhance survival of BMDCs after ischemic injury [248]. Porous alginate cryogels, a synthetic niche, can enhance the paracrine effects of MSCs [249]. Moreover, the use of biomimetic macroporous polyethylene glycol hydrogel is an effective method to significantly promote the multiplication of HSCs before transplantation in vitro by mimicking the natural microenvironment of HSCs [250].

### Bioengineering methods

Bioengineering may be a potential strategy for replacing injured kidneys in the future. Kitamura et al. found that kidney stem/progenitor cells in the S3 segment are able to reconstitute a three-dimensional nephron-like structure in vitro [72]. Moreover, there are three main protocols to induce human kidney organoids formation by kidney progenitor cells from ESCs or induced pluripotent stem cells (iPSCs) [251]. The first protocol, put forward by Taguchi et al., who were inspired by the analysis of embryonic renal precursor cell populations, constructs kidney progenitor cell-based kidney organoids by ESCs or iPSCs [252]. The Taguchi protocol induces ESCs or iPSCs into kidney progenitor cells, which then generates kidney tubules and glomerulus-like structures, and are efficiently vascularized after transplantation [253]. The second protocol, called the Takasato protocol, uses a two-dimensional induction of kidney progenitor cells, followed by three-dimensional culture, generating kidney organoids which contains nephrons, collecting ducts, and interstitium, as well as endothelial cells, based on the adoption of ESCs or iPSCs. The renal organoids exhibit absorptive capacity for dextran [254–256]. The third protocol, the Morizane protocol, like the Takasato protocol, is divided into two-dimensional and three-dimensional steps, but requiring less time, to construct organoids containing epithelial nephron-like structures [257]. ESCs/iPSC-derived functional kidney organoids, which can be derived from patients’ own cells, present great potential for kidney replacement therapies in the future [258]. Nevertheless, there are many challenges that need to be overcome before the application of kidney organoids in humans, including strategies to improve the scalability and vascularization of organoids. Moreover, it has been found that cells in kidney organoids are much more immature than cells in the adult kidney, and there are off-targeted non-renal cells within organoids [251].

The application of a decellularized extracellular matrix (dECM) scaffold, providing a 3D environment mimicking the natural tissues. The forms of which includes gels, patches, sections, blocks, and coatings, will also play a significant role in regenerative medicine and bioengineering in the future [242]. dECM scaffolds from kidney, in which there are no cells, or important cell-associated immunogenic markers, but only a native renal architecture and extracellular matrix protein, create a niche similar to the natural renal tissues, facilitate the recruitment of stem/progenitor cells, enhance neovascularization, and promote restoration of kidney function [259, 260]. dECM scaffolds from porcine, preserving the native renal architecture, extracellular components, and an intact vasculature network, may be a promising platform for kidney bioengineering due to the kidney deficiency for replace treatment [261]. SDS-treated dECM scaffolds from porcine show no cytotoxicity to primary human renal cells and depressed immunoreactivity by the thorough clearance of porcine cellular material [262]. A study shows that after implantation of the porcine dECM scaffold into the porcine kidney, the scaffold is easily reperfused, can sustain blood pressure, and is tolerated during the study period without blood extravasation. However, inflammatory cells and complete thrombosis can also be observed [263]. In a study, the researchers plant mouse ESCs in dECM scaffolds of rat kidney to induce recellularization and organoid construction in vitro, and then implant it into a uninephrectomized rat. The result shows that these recellularized scaffolds are easily reperfused, could tolerate blood pressure, and produce urine with no blood leakage for approximately 2 weeks [264]. Although the regeneration of functional whole organs has not been accomplished and there are many obstacles still need to be overcome, the combination of stem/progenitor cells and dECM scaffolds will hopefully overcome these challenges one day and take an advance in regenerative medicine [265].

## Conclusions

There is a range of stem/progenitor cells, including kidney-resident stem/progenitor cells in the different areas of the kidney, with their own characteristics, and those that are derived from bone marrow and then home to the kidney. After kidney injury, these stem/progenitor cells can migrate into injured areas through a complicated mechanism, where they exert a protective effect on the inflammatory and hypoxic microenvironment of the injured kidney through differentiation or paracrine functions.

**Fig. 3:**
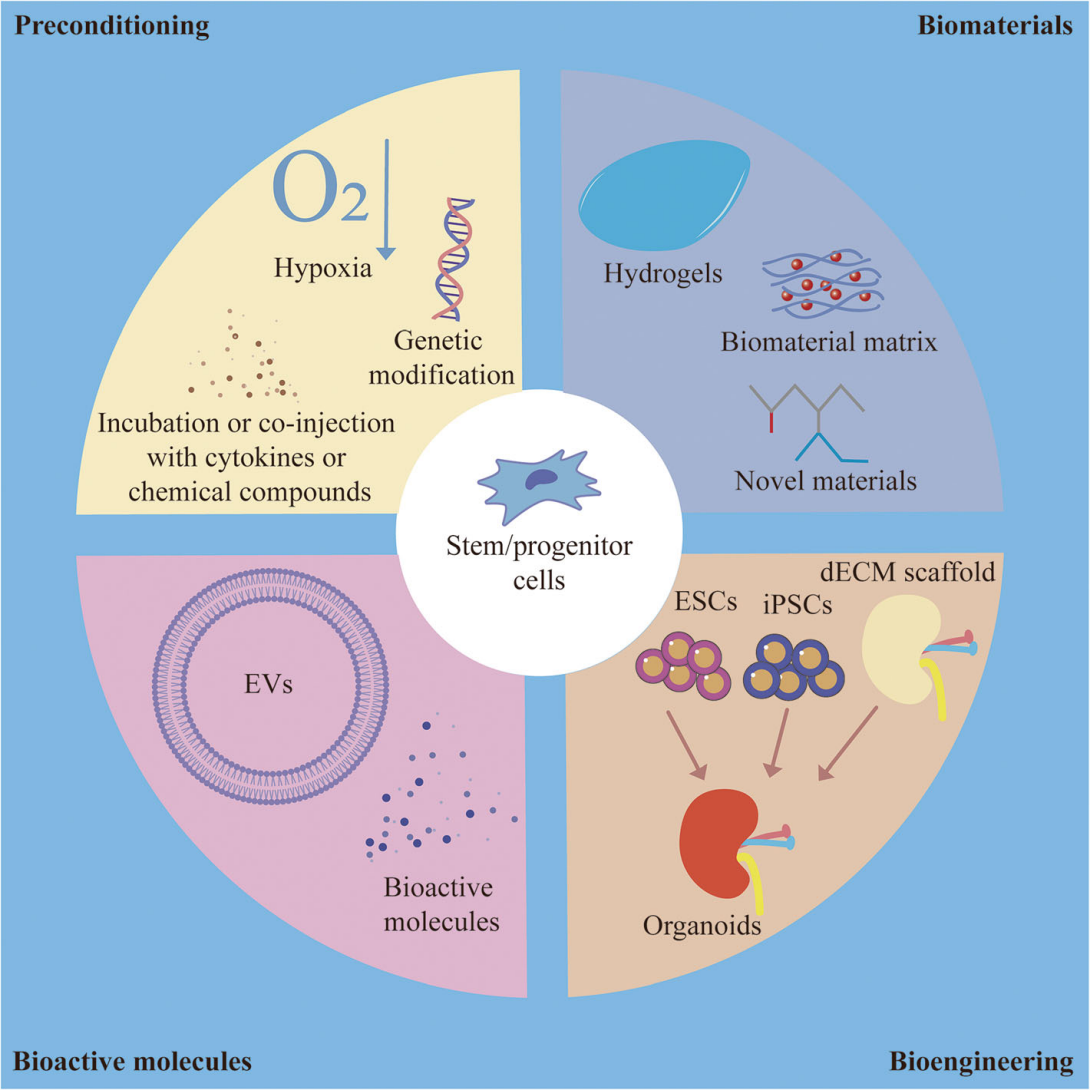
There are four potential strategies for improving the therapeutic effects of stem/progenitor cell-based therapy, including preconditioning, application of biomaterials, bioactive molecules, and bioengineering. Preconditioning mainly includes hypoxia, genetic modification, and administration with cytokines or chemical compounds. Biomaterials include hydrogels, biomaterial matrix, and other novel materials. The application of the bioactive molecules and EVs secreted by stem/progenitor cells is also helpful. And the application of dECM scaffold and ESCs or iPSCs to regenerate a functional whole organ is a prospective strategy in the future

There are some appropriate and promising strategies for stem/progenitor cell-based therapies (Fig. 3). Stem cell preconditioning is an effective strategy to improve stem cell homing and survival, so as to enhance their kidney protective effect. However, the stem/progenitor cells’ function is impaired in CKD patients, leading to the unsatisfactory therapeutic effects, but allogenic stem/progenitor cells may be rejected. The application of bioactive molecules secreted by stem/progenitor cells could overcome this challenge. Because the combination with biomaterials can overcome the rapid clearance of stem/progenitor cells and their bioactive products in vivo, enhance their renal selectivity, and provide a welcome microenvironment to promote their survival and function, this strategy should also be taken into consideration. Finally, with the potency to biotechnological generation of a functional whole kidney in the future, a bioengineering method may be a promising future prospect.

## Data Availability

All data are included in this published article.

## Abbreviations

AKI: Acute kidney injury
bFGF: Basic fibroblast growth factor
BMDCs: Bone marrow-derived stem/progenitor cells
BMP-7: Bone morphogenetic protein 7
BMSCs: Bone marrow mesenchymal stem cells
CKD: Chronic kidney disease
CXCR4: Chemokine (C-X-C motif) receptor 4
dECM: Decellularized extracellular matrix
EGFP: Enhanced green fluorescent protein
EPCs: Endothelial progenitor cells
ESCs: Embryonic stem cells
ESRD: Stage of renal disease
EVs: Extracellular vesicles
FSGS: Focal segmental glomerulosclerosis
HA: Hyaluronic acid
HIF: Hypoxia-inducible factor
HGF: Hepatocyte growth factor
HSCs: Hematopoietic stem cells
IFN-γ: Interferon-gamma
IGF-1: Insulin-like growth factor-1
IRI: Induced pluripotent stem cells, ischemia/reperfusion injury
iPSCs: Induced pluripotent stem cells
MSCs: Mesenchymal stem cells
PDGF: Platelet-derived growth factor
Sca-1: Stem cell antigen-1
SDF-1: Stromal-derived factor-1
SOD: Superoxide dismutase
TGF-β: Transforming growth factor β1
TNF-α: Tumor necrosis factor-alpha
UUO: Unilateral ureteral obstruction
VEGF: Vascular endothelial growth factor
